# Stacking among the clips of the poly-aromatic rings of phenazine with hydroxy-aromatics and photophysical properties†

**DOI:** 10.1039/c9ra07602f

**Published:** 2019-10-17

**Authors:** Rinki Brahma, Munendra Pal Singh, Jubaraj B. Baruah

**Affiliations:** Department of Chemistry, Indian Institute of Technology Guwahati Guwahati 781 039 Assam India juba@iitg.ac.in

## Abstract

Clip-like arrangements of molecules in the cocrystals of phenazine with hydroxy-aromatics in their respective self-assemblies and photophysical properties were presented. Phenazine cocrystals with 1,2-dihydroxybenzene provided assembly with butterfly-like arrangements. In these cocrystals, the phenazine molecules occurred in parallel pairs having extensive π-stacking. The clip-like cocrystals with 1,3-dihydroxybenzene also exhibited parallel pairs of phenazine molecules that were parallel cofacial π-stacked. The hydrated cocrystals of phenazine with 1,2,3-trihydroxybenzene had chains of parallel cofacial phenazine rings having three distinguishable π-separation distances among the centroids of the phenazine rings. Also, 2,7-dihydroxynaphthalene formed a clip-like cocrystal with phenazine, which encapsulated an additional molecule of phenazine. This cocrystal also provided chain-like parallel arrangements of the phenazine molecules. The emission and quantum yields of the cocrystals were determined by the integrating sphere method, which indicated that only the cocrystal of phenazine with 2,7-dihydroxynaphthalene showed monomer-like emission of phenazine and the rest of the cocrystals were in a quenched state. In the solution phase, quenching of the emission of hydroxynaphthalene was observed when phenazine was added to an independent solution of 2,7-dihydroxynaphthalene or another hydroxynaphthalene. However, when hydroxybenzenes were added to a solution of phenazine, fluorescence enhancements of phenazine occurred due to photo-electron transfer.

## Abstract 2

π-Stackings control the photoluminescence efficiencies in solids, whereas in solutions, the ON or OFF processes are dependent on the hydroxyaromatics.

## Graphical abstract

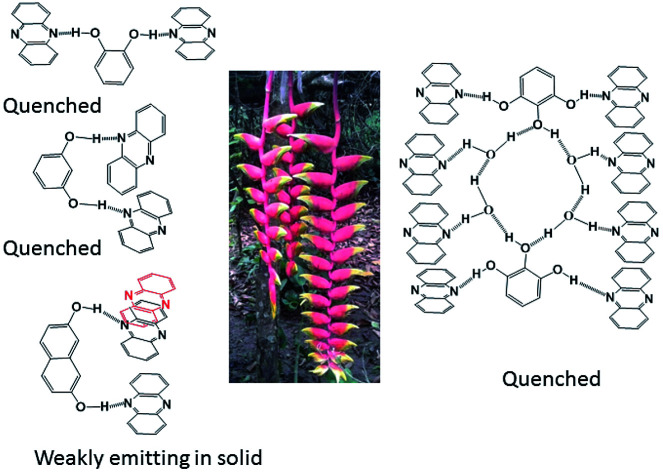

## Introduction

**Fig. 1:**
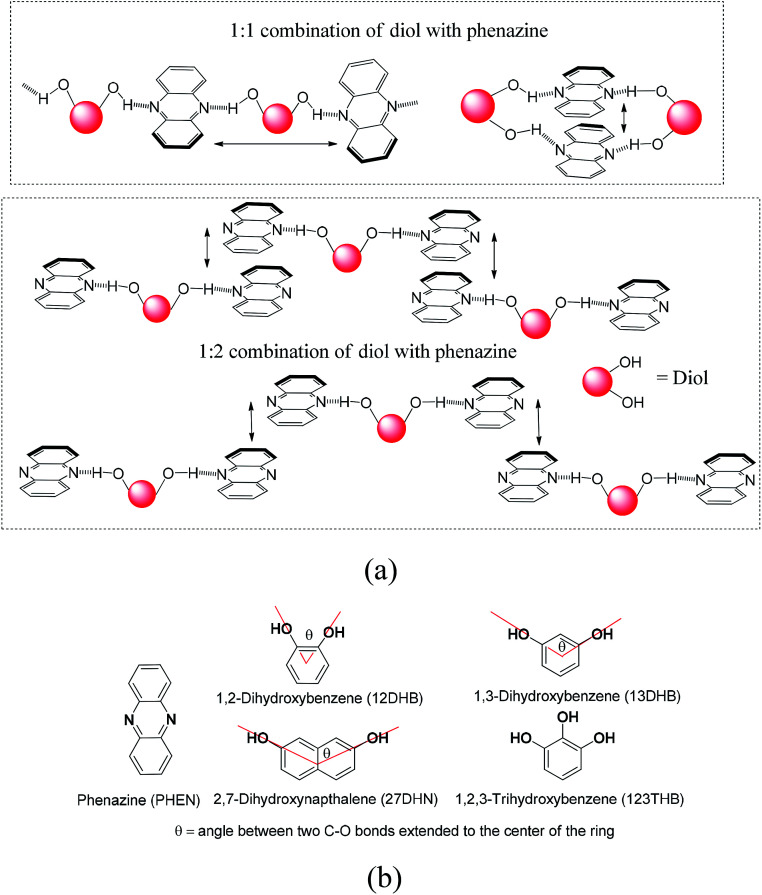
(a) Some possible hydrogen-bonded assemblies of phenazine with aromatic diols and (b) structures of phenazine and different organic aromatic diols used in this study.

The new discoveries on different aspects on the consequences of weak interactions have been fuelling the quest to modulate optical properties by them in solid and solution.^1–7^ Changing weak interaction schemes in conformation polymorphs has been routinely demonstrated to change photophysical properties.^8–11^ The cocrystals and salts are among the non-covalently linked compounds, where the proton transfer^12–18^ and organized structures^19,20^ change the orientations of fluorophores by interactions with partner molecules. The π-interactions have an immense role in photophysical properties,^21–23^ ion-molecular recognition,^24–26^ and catalytic reactions.^27^ These interactions are common in poly-aromatic compounds to guide the mechanism of photoluminescence processes.^28–30^ Stacked arrangements through π-interactions contribute to dimer- and excimer-like emissions^31–34^ and Dexter quenching.^35^ Different types of aggregates where different packings through the C–H⋯π interactions or π–π interactions are present show distinguishable features.^36–43^ The solvent molecules within lattices are of concern in discerning optical properties.^44^ Thus, the structures of self-assemblies are of general concern in the elucidation of photoluminescence properties. The dimer-like emissions^45–47^ and Förster resonance transfer emission^48^ require designs to hold the fluorophores in close proximity in definite orientations. We chose to study the self-assemblies of phenazine with different aromatic diols in anticipation that the position and stoichiometry would guide the stacking pattern.^49,50^ For example, a diol forming an adduct with an equimolar ratio may form a linear polymeric structure or cyclic-oligomers; in contrast, a 1 : 2 ratio combination would provide discrete units, and an arrangement shown in Fig. 1a for such a composition would violate the Etter's rule. Such adducts would provide various stacking arrangements from parallel eclipsing to parallel translated arrangements, as shown in Fig. 1a. In the latter case, one will have to leave the favorable hydrogen bonding sites of phenazine free; in fact, such an observation can be possible as the exceptional adducts of poly-hydroxyaromatics violating the Etter's rule have been described with other hosts.^51^ The π-stacking among the phenazine rings or dihydroxyaromatics would provide avenues to study the photoluminescence properties in the solid state. This is anticipated, as fluorophores such as anthracene π-stacked in different ways operate with different emission mechanisms.^52,53^ With the above points in mind, we studied the structures and photoluminescence properties of the cocrystals of the diols and triols listed in Fig. 1b.

## Experimental

### General

Infrared spectra of the solid samples were recorded on a PerkinElmer Spectrum-One FT-IR spectrophotometer by making KBr pellets. Powder X-ray diffraction patterns were recorded using a Bruker powder X-ray diffractometer D2 phaser. ^1^H-NMR spectra of complexes were recorded on a BRUKER Ascend-600 MHz NMR spectrometer using TMS as the internal standard. A PerkinElmer Lamda-750 spectrometer was used to record the solid-state UV-visible spectra by diffuse reflectance. Fluorescence emissions were measured in a Horiba Jobin Yvon Fluoromax-4 spectrofluorometer by taking definite amounts of solutions or definite amounts of solid samples and exciting at the required wavelength. Quantum yields were measured by taking definite amounts of solids and solutions and exciting at the required wavelength in a Horiba Jobin Yvon Fluoromax-4 spectrofluorometer by Petite integrating sphere method. The fluorescence emission (*E*_c_) and the scatter (*L*_c_) for the sample and blank (*L*_a_ and *E*_a_) were recorded. From these spectral measurements (sample and blank), the quantum yields were calculated by using the equation *φ* = [(*E*_c_ − *E*_a_)/(*L*_a_ − *L*_c_)].

### Preparation and spectroscopic features of the cocrystals

The cocrystals of 12DHB and 13DHB with phenazine were obtained by slow evaporation of a solution of phenazine (360 mg, 2 mmol) with the respective coformer (110 mg, 1 mmol) in methanol (20 ml) carried out at room temperature. After about 30 h, crystals appeared, which were decanted and collected for characterization. Similarly, the cocrystals of 27DHN and 123THB with phenazine were obtained from crystallization by slow evaporation at 27–30 °C of the respective methanol (20 ml) solution containing phenazine (450.5 mg, 2.5 mmol) with 27DHN (160.2 mg, 1 mmol) or 123THB (126.1 mg, 1 mmol). In these cases also, after about 25–30 h, the solution was reduced to about 5 ml and the cocrystals were formed as exclusive crystalline products. We used only the pure crystals for study and did not use the bulk of the completely evaporated product as it carries starting components. 2(Phen)·12DHB: isolated yield of crystals: 62%. ^1^H-NMR (600 MHz, DMSO-d_6_): 8.82 (s, 2H), 8.27 (m, *J* = 6 Hz, 4H), 7.99 (m, *J* = 6 Hz, 4H), 6.72 (t, *J* = 6 Hz, 2H), 6.60 (d, *J* = 6 Hz, 2H). IR (KBr, cm^−1^): 3446 (br), 1623 (m), 1589 (w), 1514 (s), 1468 (m), 1364 (s), 1248 (s), 1192 (s), 1141 (w), 1094 (s), 1038 (m), 911 (m), 848 (s), 743 (s), 595 (m). 2(Phen)·13DHB: isolated yield of crystals: 69%. ^1^H-NMR (600 MHz, CDCl_3_): 8.27 (m, *J* = 6 Hz, 8H), 7.86 (m, *J* = 6 Hz, 8H), 7.09 (t, *J* = 6 Hz, 1H), 6.42 (d, *J* = 6 Hz, 2H), 5.74 (s, 1H), 3.49 (s, 1H). IR (KBr, cm^−1^): 3449 (br), 2882 (w), 1597 (s), 1515 (s), 1472 (s), 1214 (s), 1178 (s), 1143 (s), 906 (m), 840 (m), 745 (s), 694 (w). 2.5(Phen)·27DHN: isolated yield of crystals: 71%. ^1^H-NMR (600 MHz, DMSO-d_6_): 9.67 (s, 2H), 8.23 (s, 4H), 7.95 (s, 4H), 7.58 (d, *J* = 12 Hz, 2H), 6.87 (s, 2H), 6.82 (d, *J* = 12 Hz, 2H). IR (KBr, cm^−1^): 3453 (br), 3049 (m), 1621 (s), 1602 (w), 1531 (m), 1514 (s), 1463 (s), 1432 (m), 1379 (s), 1279 (m), 1239 (m), 1198 (s), 1158 (m), 1142 (m), 1118 (m), 859 (s), 742 (s). 2.5(Phen)·123THB. 2H_2_O: isolated yield of crystals: 58%. ^1^H-NMR (600 MHz, DMSO-d_6_): 8.27 (m, *J* = 6 Hz, 4H), 7.86 (m, *J* = 6 Hz, 4H), 6.63 (t, *J* = 6 Hz, 1H), 6.49 (d, *J* = 6 Hz, 2H). IR (KBr, cm^−1^): 3476 (br), 1629 (m), 1532 (w), 1514 (s), 1469 (s), 1433 (s), 1365 (m), 1326 (m), 1248 (m), 1204 (m), 1143 (m), 1116 (m), 1065 (m), 1011 (s), 907 (m), 823 (s), 745 (s).

### Crystal structure determination

Single-crystal X-ray diffraction data for cocrystals 2(Phen)·12DHB and 2.5(Phen)·123THB·2H_2_O were collected at 296 K with Mo Kα radiation (*λ* = 0.71073 Å) by a Bruker Nonius SMART APEX CCD diffractometer equipped with a graphite monochromator and an Apex CCD camera, and those for the other two cocrystals were collected on an Oxford SuperNova diffractometer. Data reductions and cell refinement for the Oxford SuperNova diffractometer were performed by CrysAlisPro software and for Bruker Nonius SMART APEX CCD diffractometer, they were performed using SAINT and XPREP softwares. Structures were solved by direct methods and were refined by full-matrix least-squares on *F*^2^ using the SHELXL-2014 software. All non-hydrogen atoms were refined in anisotropic approximation against *F*^2^ of all reflections. Hydrogen atoms were placed at their geometric positions by riding and refined in the isotropic approximation. The crystallographic parameters are listed in Table 1.

**Table 1:** Crystallographic parameters of the cocrystals of phenazine

Parameters	2(Phen)·12DHB	2(Phen)·13DHB	2.5(Phen)·123THB·2H_2_O	2.5(Phen)·27DHN
Formula	C_30_H_22_N_4_O_2_	C_15_H_11_N_2_O_1_	C_72_H_60_N_10_O_10_	C_80_H_56_N_10_O_4_
CCDC no.	1946193	1946194	1946196	1946195
Mol. wt	470.51	235.26	1225.30	1221.34
Space group	*P*1̄	*C*2/*c*	*P*1̄	*P*2_1_/*c*
*a*/Å	7.403(3)	14.2921(9)	9.7943(10)	9.382(6)
*b*/Å	17.584(6)	11.4224(7)	12.0978(12)	18.747(12)
*c*/Å	18.374(6)	14.4820(10)	13.8839(14)	17.729(11)
*α*/deg	76.445(11)	90	89.390(3)	90
*β*/deg	79.816(12)	102.490(6)	78.850(3)	97.799(7)
*γ*/deg	79.751(12)	90	72.021(3)	90
*V*/Å^3^	2265.1(14)	2308.2(3)	1533.1(3)	3089(3)
Density/g cm^−3^	1.380	1.354	1.327	1.313
Abs. coeff./mm^−1^	0.089	0.087	0.090	0.083
*F*(000)	984	984	642	1276
Total no. of reflections	7999	2036	5429	5457
Reflections, *I* > 2*σ*(I)	4744	1417	3512	2434
Max. *θ*/degree	25.047	25.042	25.048	25.048
Ranges (*h*, *k*, *l*)	−8 ≤ *h* ≤ 8	−11 ≤ *h* ≤ 16	−11 ≤ *h* ≤ 11	−11 ≤ *h* ≤ 11
	−20 ≤ *k* ≤ 20	−13 ≤ *k* ≤ 9	−14 ≤ *k* ≤ 14	−22 ≤ *k* ≤ 22
	−21 ≤ *l* ≤ 21	−17 ≤ *l* ≤ 16	−16 ≤ *l* ≤ 16	−21 ≤ *l* ≤ 21
Complete to 2*θ* (%)	99.9	100.0	99.9	99.6
Data/restraints/parameters	7999/0/653	2036/0/165	5429/2/429	5457/0/426
GooF (*F*^2^)	1.126	1.075	1.064	1.073
*R* indices [*I* > 2*σ*(I)]	0.0828	0.0503	0.0631	0.0596
w*R*_2_ [*I* > 2*σ*(I)]	0.2413	0.1201	0.1966	0.1247
*R* indices (all data)	0.1310	0.0732	0.0956	0.1491
w*R*^2^ (all data)	0.2413	0.1380	0.2352	0.1662

## Results and discussion

Four cocrystals that were studied were the cocrystals of phenazine with 1,2-dihydroxybenzene (2 : 1); 1,3-dihydroxybenzene (2 : 1), 2,7-dihydroxynaphthalene (2.5 : 1) and 1,2,3-trihydroxybenzene (2.5 : 1); all of them were prepared through solution crystallization. The cocrystals were anhydrous except the one formed with 1,2,3-trihydroxybenzene, which was a hydrate. Our interest was the π-interacting system without the interference of third foreign molecules. Thus, we chose three independent anhydrous cocrystals having different projections of the hydroxyl groups in their respective rings and also dealt with the hydrated cocrystal of phenazine with 1,2,3-trihydroxybenzene. The cocrystals were characterized by recording their ^1^H-NMR, IR, and powder-XRD spectra and by determining the single-crystal structures.

**Fig. 2:**
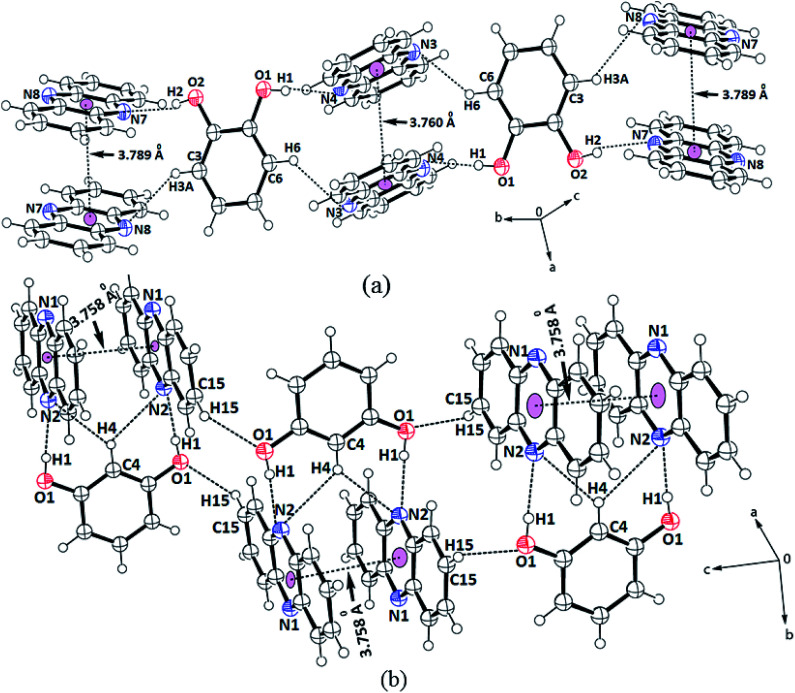
Portions of the self-assembly of (a) 2(Phen)·12DHB, (b) 2(Phen)·13DHN.

The structures helped us delineate the stacking patterns between the phenazines or between the diols or both; hence, only the relevant portion of the structures relating these issues was presented. The portions of packing showing the π–π interactions of the cocrystals are shown in Fig. 2. In this 2 : 1 cocrystal of phenazine with 1,2-dihydroxybenzene, the latter part serves as a bridge to hold two phenazine molecules through O2–H⋯N7 and O1–H⋯N4 interactions (Fig. 2a) to form a propeller-like structure. The cocrystal self-assembled through C3–H⋯N8 and C6–H⋯N3 interactions (*d*_D⋯A_ = 3.368(5) Å, ∠D–H⋯A = 140° and *d*_D⋯A_ = 3.316(5) Å, ∠D–H⋯A = 142°, respectively), forming linear chain-like arrangements, as illustrated in Fig. 2a. The prominent hydrogen bond parameters are listed in Table 1S.† The phenazine rings were π-stacked and the centroid-to-centroid distance of the parallel phenazine rings eclipsing each on either side of the 1,2-dihydroxybenzene ring was different. The distance of separation on one side was 3.760 Å, whereas on the other side, it was 3.789 Å. Generally, self-assemblies possessing adjacent aromatic rings in parallel cofacial, parallel displaced or edge-to-face orientations get involved in π-interactions.^54^ The centroid-to-centroid distance of up to 3.5 Å was suitable to have such interactions, whereas the distance within 3.7–3.8 Å for parallel displaced and cofacial parallel arrangements of rings was established for weak interactions.^55^ Hence, the observed centroid-to-centroid distances among the cofacial parallel rings of the phenazine molecules revealed that there were very weak π-interactions among such rings located on either side of 1,2-dihydroxybenzene. In the ^1^H-NMR spectrum recorded in DMSO-d_6_, the aromatic protons appear at similar chemical shift positions to that of the parent components but the chemical-shift positions of the O–H protons are affected showing the existence of hydrogen bonds. In the solid-state IR spectrum, the OH stretching appears as a broad peak at 3446 cm^−1^. As the powder XRD pattern of the bulk of crystals shows no other phase and the crystals obtained are an identical invariant of stoichiometry, it can be confirmed that in solution, the 2 : 1 cocrystal was exclusively formed from phenazine with 1,2-dihydroxybenzene in the methanol solution.

Phenazine also formed a 2 : 1 cocrystal with 1,3-dihydroxybenzene, which also has a similar feature in holding the two molecules of phenazine by moderate hydrogen bonds (Fig. 2b). The self-assemblies were different due to the difference in the position of the OH groups on the ring. The C–H bond located in between the two hydroxyl groups of the diol was involved in very weak bifurcated C–H⋯N interactions (*d*_D⋯A_ = 3.316(5) Å and ∠D–H⋯A = 142°). These subsidiary interactions helped hold the two phenazine rings parallel to each other in pairs with a distance of separation of 3.758 Å; this distance permits very weak π–π interactions.^55,56^ The N1 atoms were not involved in the hydrogen bonds as all good hydrogen bonding atoms did not participate in the hydrogen bonds in self-assembly. Hence, the cocrystal is an exception not following the Etter's rule. This exception occurred due to the stacking orientations that provided a tight-packed structure, so that the nitrogen atoms did not directly face each other or they were placed in orientations that were not in close vicinity or suitable to form a hydrogen bond with the donor. Such examples are often observed in closed-packed structures having a high π-stacking environment.^57–59^

**Fig. 3:**
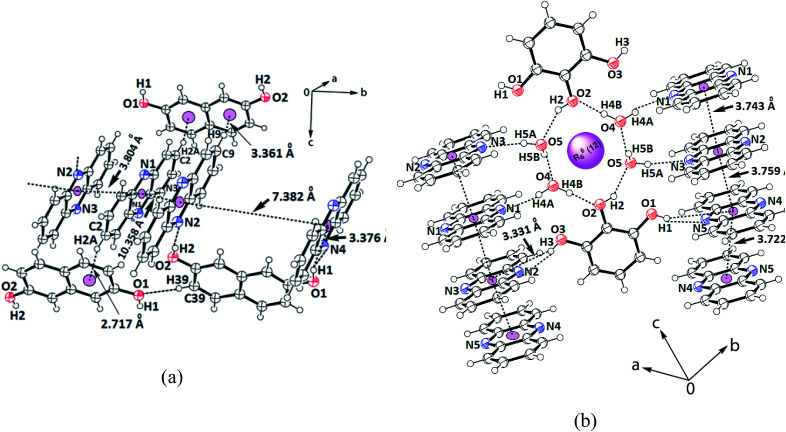
Portions of the self-assembly of (a) 2.5(Phen)·27DHN; (b) 2.5(Phen)·123THB.2H_2_O showing the π-interactions among the phenazine rings.

The 2,7-dihydroxynaphthalene cocrystal had a 2.5 : 1 molar ratio. In this example, two hydroxy groups of each 2,7-dihydroxynaphthalene could hold two independent phenazine molecules through O1–H⋯N4 and O2–H⋯N2 hydrogen bonds (Table S1†), forming a clip-like structure (Fig. 3a). A third molecule of phenazine was placed over one of the phenazine molecules directly hydrogen bonded to 2,7-dihydroxynaphthalene. Thus, there were three molecules of phenazines that were arranged as stacked molecules together in a repeated manner. The hydrogen-bonded clips were arranged in the opposite direction. The encapsulated phenazine molecule was located in between two adjacent clips. This phenazine molecule actually became confined within the self-assembly as it provided a bridge between two independent clip-like structures located at its two ends through C–H⋯π interactions. In other words, the two rings of 2,7-dihydroxynaphthalene of the clips were involved in the C9–H⋯π_(C38)_ and C2–H⋯π_(C31–36)_ interactions with this encapsulated phenazine molecule. In the overall assembly, phenazine molecules were located at parallel positions on the top and bottom of the guest. The eclipsed phenazine molecules at parallel positions exhibited very weak facial π–π interactions on one side but on the other side, there were no facial π–π interactions. The π-separations between phenazine and the hydroxynaphthalene neighbors in parallel positions were 3.804 Å and 10.358 Å. Hence, out of these three phenazine molecules, one is far away to have π-interactions and it may be considered as an isolated phenazine molecule. The other three cocrystals had relatively shorter centroid-to-centroid distances to have cofacial parallel stacked π-interactions between the phenazine molecules than such interactions in the cocrystal of phenazine with 27DHN. The cocrystal of 1,2-dihydroxybenzene did not have clips similar to the one observed in the cocrystals of phenazine with 1,3-dihydroxybenzene or 2,7-dihydroxynaphthalene. Due to the outward projection of the O–H bonds of 1,2-dihydroxybenzene participating in hydrogen bonds with two phenazine molecules, the cocrystal of phenazine with 1,2-dihydroxybenzene exhibited a flattened structure. This provided a butterfly-like structure. Phenazine has structural similarity with acridine; the orderly structures of acridine are observed in salts having carboxylic acid. These assemblies encapsulate polyaromatic guest molecules.^59^ However, we have a relatively simple cocrystal between 2,7-dihydroxynaphthalene with phenazine in a 1 : 2.5 ratio, where an additional phenazine molecule gets encapsulated. Polymorphs of host–guest complexes formed by naphthalimide-functionalized carboxylic acids with quinoline encapsulating one of the components, namely, quinoline were reported earlier by us.^60^ Comparing these examples, the cocrystal of 2,7-dihydroxynaphthalene with phenazine is an exception to contradict the Etter's rule.^61^

Finally, the self-assembly of the hydrated cocrystal of 1,2,3-trihydroxybenzene (common name pyrogallol) comprises *R*_6_^6^(12) synthons. The synthon has two trihydroxybenzene molecules and four water molecules, forming a hydrogen-bonded cyclic structure (Fig. 3b). This cocrystal comprises one molecule of 1,2,3-trihydroxybenzene with 2.5 molecules of phenazine and two molecules of water. To form self-assembly, the two water molecules are linked together at the two sides of two intervening 1,2,3-trihydroxybenzene molecules. The centrally located O–H bond (that is at the 2-position of the 1,2,3-trihydroxybenzene ring) is involved in the bifurcated hydrogen bonds with those water molecules. Each water molecule of this assembly has one O–H bond free to serve as a hydrogen bond donor. The four phenazine molecules form an O–H⋯N hydrogen bond with them and get anchored to remain parallel to each other but perpendicular to the synthon. On the other hand, the two O–H groups located at the 1 and 3-positions of the ring of trihydroxybenzene are not a part of the *R*_6_^6^(12) synthon; they project away from the synthon.^62^ These two hydroxyl groups are hydrogen-bonded to the phenazine molecules through the O–H⋯N bonds, where the O3–H⋯N2 bond has *d*_D⋯A_ = 2.757(3) Å, ∠D–H⋯A = 163° and O1–H⋯N5 has *d*_D⋯A_ = 2.787(3) Å, ∠D–H⋯A = 162°. Thus, there are parallel arrays of phenazine molecule chains along the *c*-crystallographic axis. These parallel cofacial rings are located on both sides of the cyclic synthon; they are arranged like an infinite chain of parallel rings of phenazine molecules. The distances between the centroids of the phenazine rings of the ones that are hydrogen-bonded to the O–H bonds of water molecules, the one connected to O–H of trihydroxybenzene and the ones connected to phenolic O–H at the two ends, and the ones from adjacent synthons are 3.743 Å, 3.759 Å and 3.722 Å, respectively. Since the distances are >3.5 Å but the increments are in close range of this stipulated distance to have π-interactions,^52^ there are weak π-interactions among the phenazine rings. In literature, it is found that phenazine with 1,3,5-trihydroxybenzene (also known as phloroglucinol) forms different hydrate as well as anhydrous cocrystals; these four cocrystals each differing in stoichiometry or the extent of hydration were characterized.^49^ In our case, we obtained only one form of the hydrated cocrystal of phenazine with pyrogallol from solution crystallization. This result compared with the literature suggests that the positions and orientations of the hydroxy groups on the benzene ring guide the stoichiometry of the cocrystals.

**Fig. 4:**
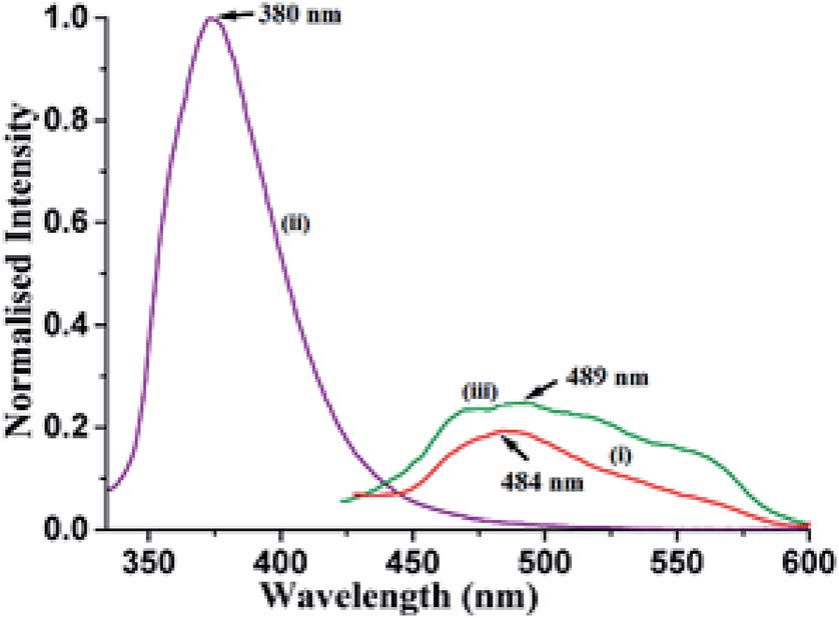
Fluorescence emission of the solid samples of (i) 2.5(Phen)·27DHN (*λ*_ex_ = 335 nm, *λ*_em_ = 484 nm); (ii) 27DHN (*λ*_ex_ = 335 nm, *λ*_em_ = 380 nm); (iii) Phen (*λ*_ex_ = 355 nm, *λ*_em_ = 489 nm).

Different self-interacting assemblies among the phenazine molecules in the self-assemblies provided a scope to have a rational approach to study the effects of stacking on photoluminescence properties. The phenazine molecule emits at 489 nm and the emission at this wavelength was not observed in any of the cocrystal. It was seen that the cocrystals of 1,2- and 1,3-dihydroxybenzene were very weakly emitting and they were like a quenched state. The cocrystal with 1,2,3-trihydroxybenzene was also weakly fluorescent, and it emitted at 415 nm, which was 74 nm lower than the phenazine emission. The cocrystal of 2,7-dihydroxynaphthalene showed very weak emission at 484 nm, which was similar to that of phenazine (Fig. 4). Among the cocrystals of the diols, this cocrystal was exceptional to have a phenazine molecule encapsulated between the π-stacks. The encapsulated phenazine molecules in the assembly were C–H⋯π bonded to the 2,7-dihydroxynaphthalene ring. All the other structures had parallel stacks of phenazine rings, but they showed negligible emission. The C–H⋯π interactions helped the phenazine molecule to remain in isolation to show a monomer-like emission of phenazine. The C–H⋯π interactions facilitated enhancement in emission in the cocrystals of naphthalene-aldoxime^63^ and in anthracene derivatives.^52^ The emission and quantum yield in the solid state of each cocrystal were determined, and they are listed in Table 2. The fluorescence life-time of the cocrystals showed bi-exponential decay profiles. The cocrystals of 1,2- and 1,3-dihydroxybenzene and 1,2,3-trihydroxybenzene showed similar profiles, with higher amounts of the molecules decaying through shorter life-times that ranged between 0.596 ns and 0.662 ns. Smaller fractions exhibited life-times in the range of 2.010–3.066 ns. On the other hand, the 2,7-dihydroxynaphthalene cocrystal showed a similar trend with much shorter lifetimes. In this case, a fraction of 75.20% followed the relatively shortened life-time of 0.351 ns and the fraction of 24.80% followed a shorter life-time path with 1.197 ns. This showed that the amounts of stable exciplexes formed in the first three cases were relatively high. Hence, quenching occurred, whereas in the case of 2,7-dihydroxynaphthalene, monomer-like emission was observed. The crystal structure of α as well as β-phenazine was devoid of effective π-stacking; the π-separations were 3.54 Å and 3.45 Å, respectively.^63,64^

**Table 2:** Absorbance and emission of solid samples of phenazine, 27DHN and cocrystals of phenazine

	*λ*_ab_ (nm)	*λ*_ex_ (nm)	*λ*_em_ (nm)	Quantum yield (*Φ*_F_)	Life-time in ns (% fraction)
2(Phen)·12DHB	425	365	438	0.0058	0.662 (80.149); 3.066 (19.851)
2(Phen)·13DHB	422	365	434, 464	0.0085	0.624 (78.274); 2.840 (21.726)
2.5(Phen)·123THB·2H_2_O	420	365	415	0.0181	0.596 (81.239); 2.010 fixed (18.761)
2.5(Phen)·27DHN	418	335	484	0.0105	0.351 (75.195); 1.197 (24.805)
Phenazine	417	335	489	0.0200	0.075 fixed (64.386); 0.988 (35.614)
2,7-Dihydroxynaphthalene	336	335	380	0.0582	6.379 (100.00)

**Fig. 5:**
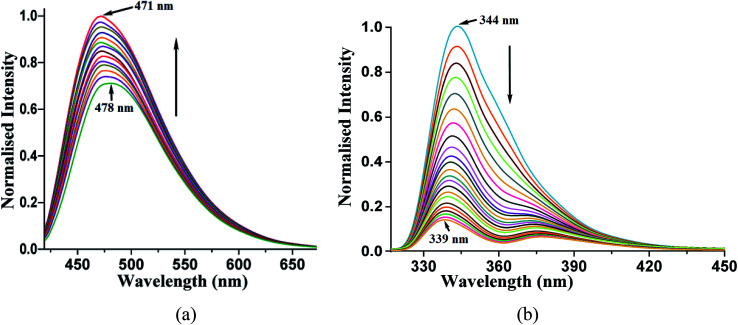
(a) Fluorescence titration (excitation at 420 nm) of phenazine (10^−3^ M in acetonitrile) by adding 1,2-dihydroxybenzene (10 μL aliquot of 10^−3^ M in acetonitrile). (b) Fluorescence titration (excitation at 280 nm) of 2,7-dihydroxynaphthalene (10^−3^ M in acetonitrile) by adding phenazine (10 μL aliquot of 10^−3^ M in acetonitrile).

A solution study enabled us to differentiate photophysical properties measured from solid samples. An acetonitrile solution of phenazine emits at 478 nm upon excitation at 420 nm. The independent titration with 1,2- or 1,3-dihydroxybenzene as well as with 1,2,3-trihydroxybenzene causes enhancement in this emission. The enhancements in the emission intensity occurred without a significant shift in the emission wavelength. A representative case of 1,2-dihydroxybenzene causing enhancement in the emission of the phenazine solution is shown in Fig. 5a. The enhancements in solutions are due to the formation of hydrogen bonds by aromatic diols with phenazine facilitating photo-electron transfer.^28^

Such enhancements in the fluorescent aromatic heterocycles by active acidic hydrogen are well known.^28^ Furthermore, the emission properties change the π-stacked aromatic molecules with slight changes in the orientations of the rings.^65^ However, in the case of 2,7-dihydroxynaphthalene interacting with phenazine, no notable increase was observed. This showed that the PET mechanism was not effective in this case. The synergic effects of aromatic stacking interactions and intrinsic acidity causing protonation helped decide the emission process.^53,66^ Hence, a reverse fluorescence titration of the 2,7-dihydroxynaphthalene solution by adding phenazine was carried out. The acetonitrile solution of 2,7-dihydroxynaphthalene emitted at 344 nm upon excitation at 280 nm. This emission peak was completely quenched upon the addition of a solution of phenazine. The Stern–Volmer plot for the changes is shown in Fig. 12S.† It shows a non-linear plot, suggesting no change in the ground state during the emission. As the concentration of phenazine increased, Dexter quenching was observed. Also, 2,7-dihydroxynaphthalene had a relatively higher quantum yield in acetonitrile (*φ* = 0.3752) than that in a solution of phenazine (*φ* = 0.1050) in acetonitrile. Hence, the quenching of the emission of 2,7-dihydroxynaphthalene was clearly seen. We checked the changes with other naphthalene derivatives such as 2-naphthol, 1,6-dihydroxynaphthalene and 1,5-dihydroxynaphthalene by adding phenazine to each solution. The Stern–Volmer plot of each case is shown in ESI (Fig. 22S†). These also showed quenching of the fluorescence emission on interactions with phenazine in solution. Among these naphthols, the lowest quenching was seen with 1,6-dihydroxynaphthalene, whereas the other three naphthols showed comparable quenching. It is clear that due to different structures, the ability of quenching of the emission of dihydroxynaphthalenes by phenazine varied with the isomers of aromatic diols.

**Fig. 6:**
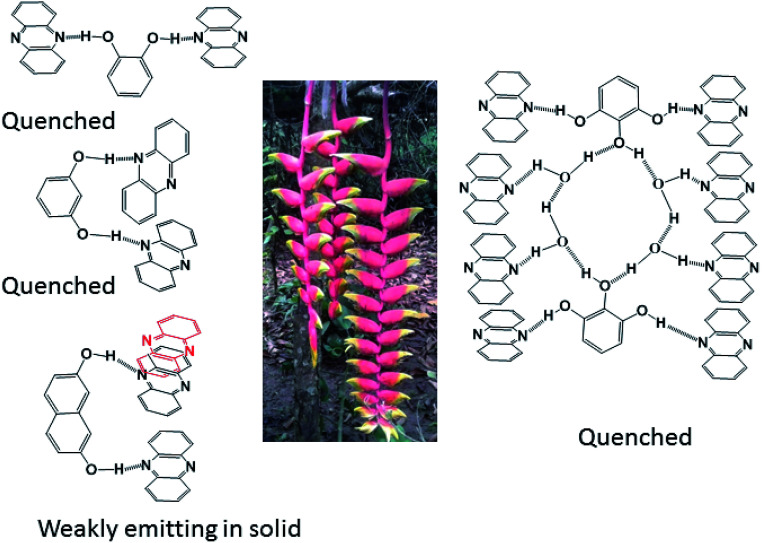
Different types of arrangements that contribute as repeating units in the self-assemblies of the four cocrystals. The central portion of the figure is on parallel and non-parallel stacks depicted by using a floral part of a plant.

From the emissive nature of the cocrystal of 2,7-dihydroxynaphthalene with phenazine in the solid state and from the observations on the quenching of the emission of 2,7-dihydroxynaphthalene in a solution on the interaction of phenazine, it was inferred that the emission processes in solids and solutions were distinguishable. In the solid state, different clip-like and chain-like structures were possible, as illustrated in Fig. 6. The structures having prominent π-stacking showed Dexter quenching in the solid state. The only exception was the cocrystal of 2,7-dihydroxynaphthalene with phenazine. This cocrystal exhibited two sets of phenazine molecules in different environments of π–π interactions. One set was involved in cofacial parallel π–π interactions and the other set was encapsulated. The encapsulated set was devoid of cofacial parallel π–π interactions but was involved in the edge-to-face C–H⋯π interactions. It was earlier suggested that the contribution of different π-interactions changes with the stacking patterns.^52^ In the same article, it was shown that the variations of cations in different salts and the edge-to-face C–H⋯π interactions help in exciplex emission to influence the intensity and position of emission. We observed a higher quantum yield in the particular case of a cocrystal of 2,7-dihydroxynaphthalene with phenazine over that of the parent phenazine in the solid state. In the cocrystal of phenazine with 2,7-dihydroxynaphthalene, the emission of 2,7-dihydroxynaphthalene at 380 nm was not observed, but it weakly emitted at 484 nm, which is a close emission wavelength to that of phenazine. This cocrystal comprised strong and weak fluorescent partners. The emission of this cocrystal at 484 nm showed that the influence of the partner molecule was prominent on the strongly emitting fluorophoric component, namely, on 27DHN, whose emission was not observed. The extent of the π–π interactions between fluorophores decreases the excited-state non-radiative relaxations, which causes partial quenching.^2^ This cocrystal did not show the emission features of the strongly emitting parent component, but it showed the emission of the weakly emitting component phenazine. This was an exceptional case as it has entrapped phenazine molecules held to 27DHN by C–H⋯π interactions; hence, emission occurred from the entrapped phenazine molecules. In solutions, molecules were not in fixed geometry; hence, the weak interactions were averaged out to show complete quenching of 2,7-hydroxynaphthalene by phenazine.

In conclusion, this study elucidated four examples of different parallel cofacial π-stacked arrangements of phenazine molecules in the cocrystals of phenazine with dihydroxyaromatics. The centroid-to-centroid distances among the parallel phenazine molecules were influenced by the directional nature of the hydrogen bonds in the respective cocrystal. Phenazine molecules encapsulated or assembled in parallel cofacial arrangements as different self-associated non-covalent assemblies such as dimers and trimers and extended chain-like parallel arrangements were observed in these self-assemblies. Depending on the centroid-to-centroid distance, the parallel cofacial π-stacks among the phenazine molecules caused complete or partial quenching of photoluminescence in the solid state. Dexter quenching was prominent in the solid state, whereas competition between photo-electron transfer emissions with Dexter quenching was observed in solutions. The emission properties in solutions exhibited wide differences from the emissions of the solid samples. In solution emissions, the ON process was observed upon the addition of hydroxybenzenes to phenazine due to photo-electron transfer, whereas the OFF emission of hydroxynaphthalenes was observed when phenazine was added to the corresponding solution of hydroxynaphthalene due to Dexter quenching.

## Conflicts of interest

The authors declare that there is no conflict of interest.

## Supplementary Material

RA-009-C9RA07602F-s001

RA-009-C9RA07602F-s002
