# Supplementary material for: Stacking among the clips of the poly-aromatic rings of phenazine with hydroxy-aromatics and photophysical properties
Source: RSC Adv. 2019 Oct 17;9(57):33403–12. doi: 10.1039/c9ra07602f (PMC9073320; doi:10.1039/c9ra07602f)
Supplement: RA-009-C9RA07602F-s001 [file RA-009-C9RA07602F-s001.pdf]

Electronic Supplementary Material (ESI) for RSC Advances.
This journal is © The Royal Society of Chemistry 2019

Stacking among the clips of poly-aromatic rings of phenazine with hydroxy-aromatics and photophysical properties

Rinki Brahma, Munendra Pal Singh, Jubaraj B. Baruah*

Supporting Information

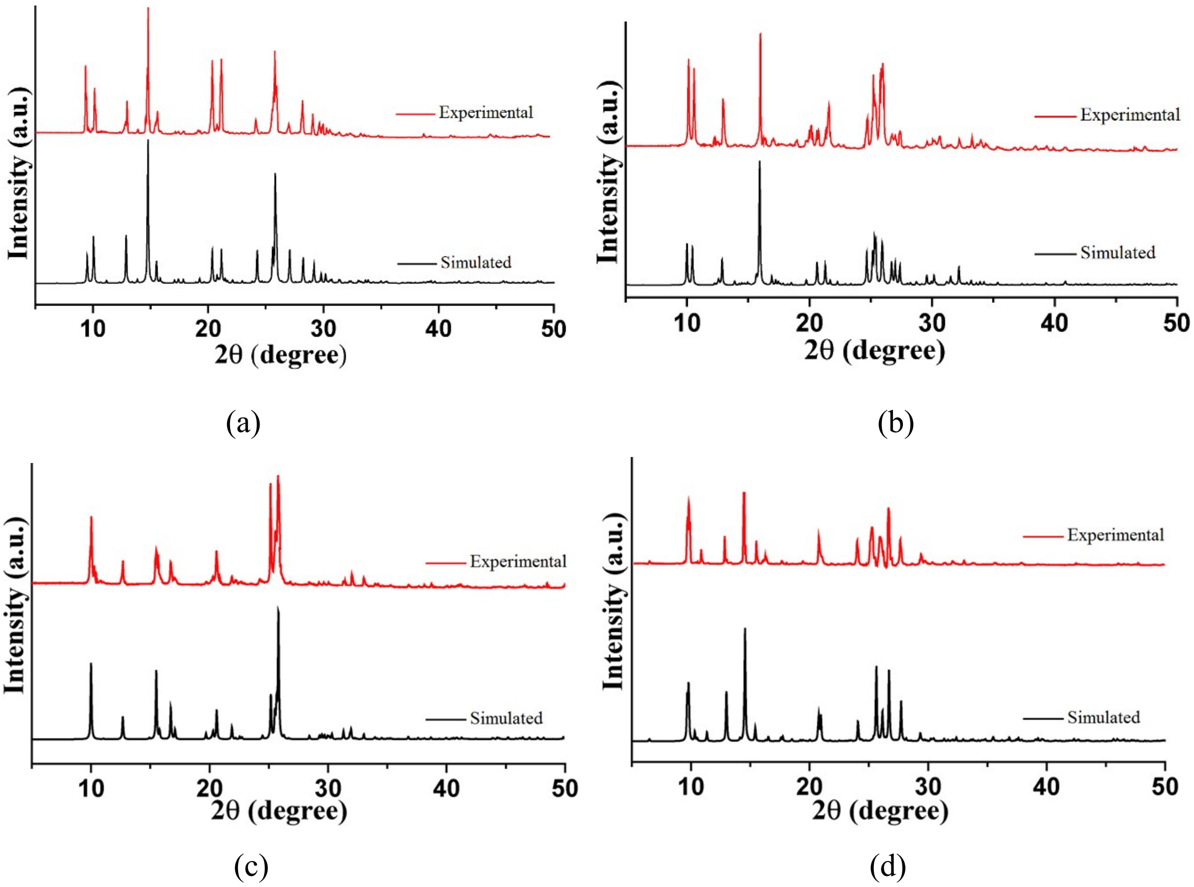

Figure 1S: PXRD patterns of cocrystals (a) 2.5(Phen).27DHN (b) 2(Phen).12DHB (c) 2(Phen).13DHB (d) 2.5(Phen)(123THB)2H_2O and simulated patterns generated from CIF file (Red= Experimental, Black= Simulated).

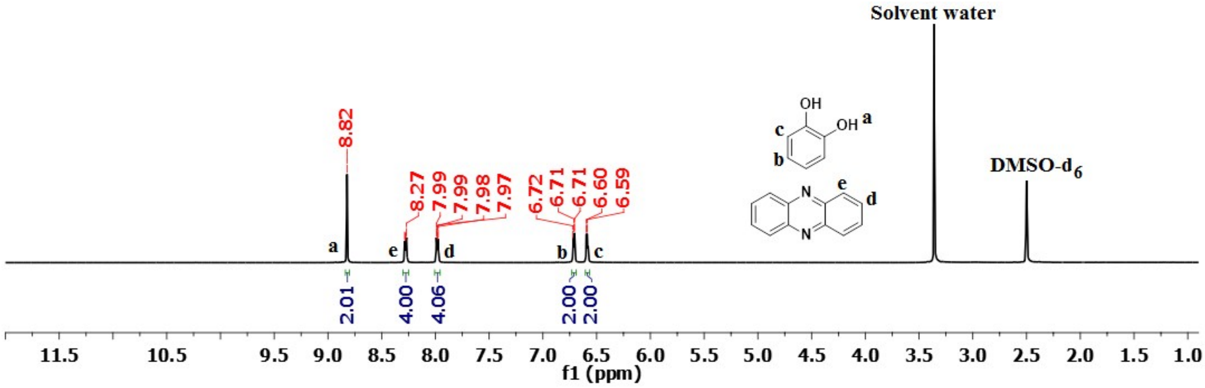

Figure 2S: ^{1}H-NMR (600 MHz, DMSO-d_6) spectra of Phen.12DHB.

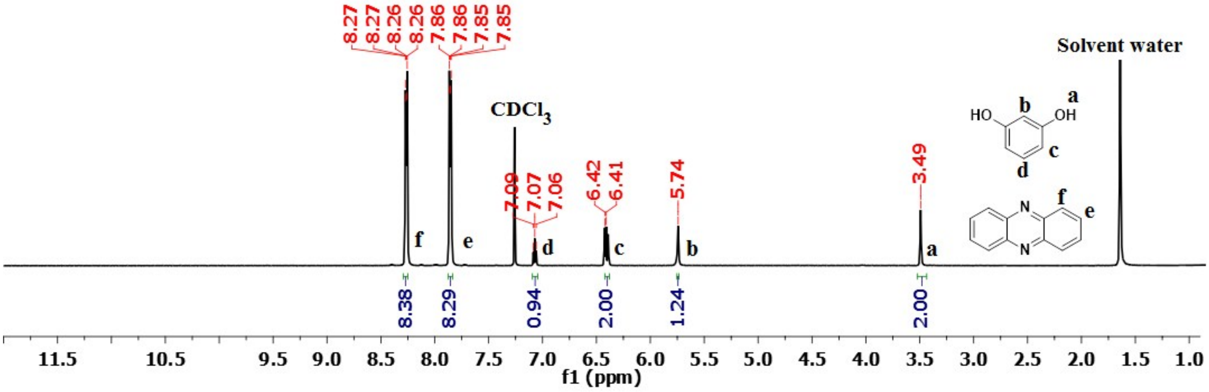

Figure 3S: ^{1}H-NMR (600 MHz, $CDCl_3$) spectra of 2(Phen).13DHB.

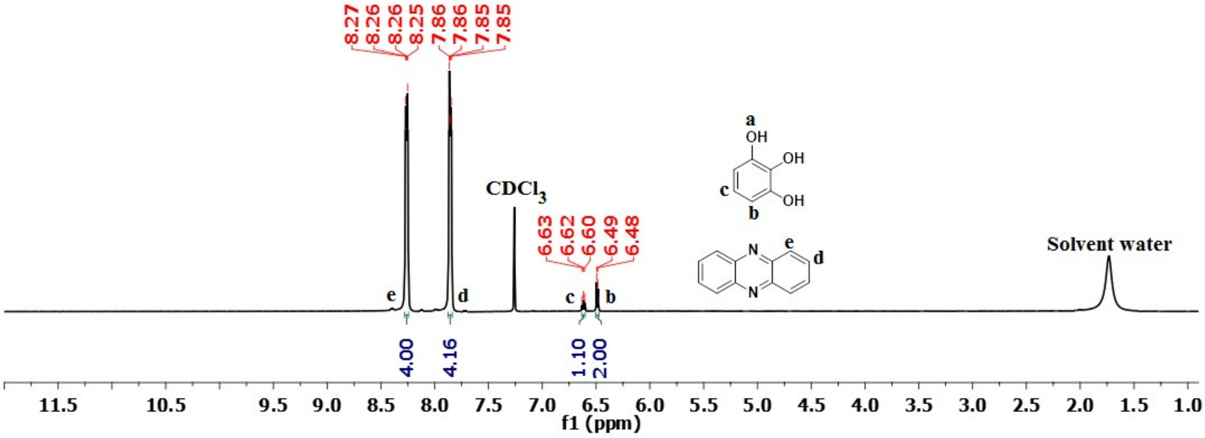

Figure 4S: ^{1}H-NMR (600 MHz, $CDCl_3$) spectra of Phen.123THB.2H_2O.

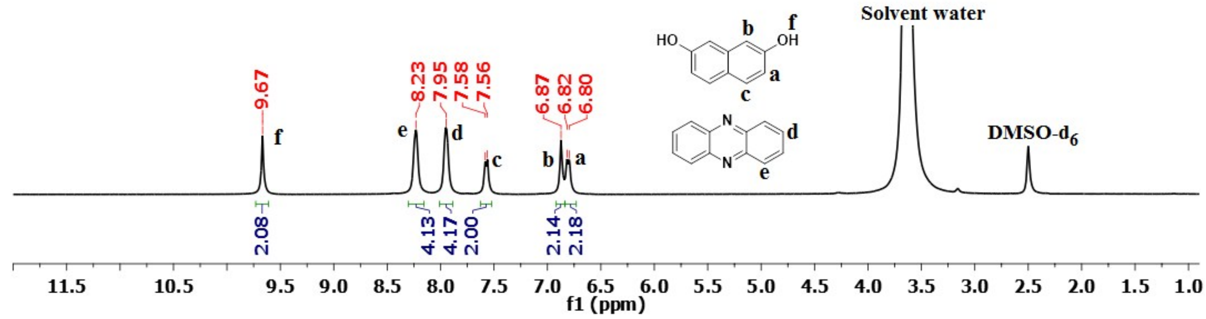

Figure 5S: ^{1}H-NMR (600 MHz, DMSO-d_6) spectra of Phen.27DHN.

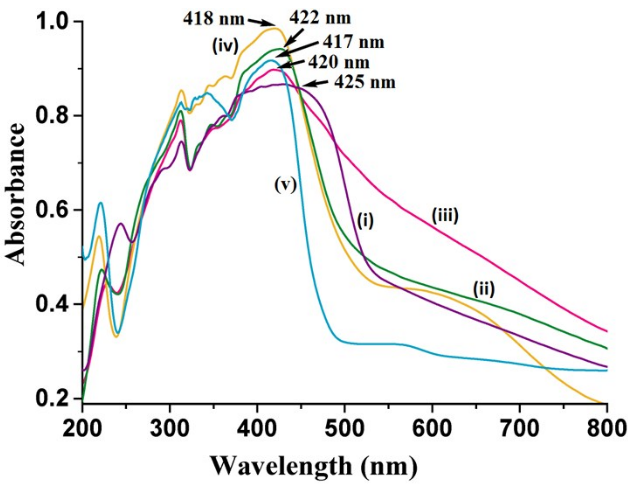

Figure 6S: UV-vis spectra of solid samples of (i) 2.5(Phen).123THB.2H_2O, (ii) 2 (Phen).12DHB, (iii) 2(Phen).13DHB, (iv) 2.5(Phen).27DHN and (v) Phenazine.

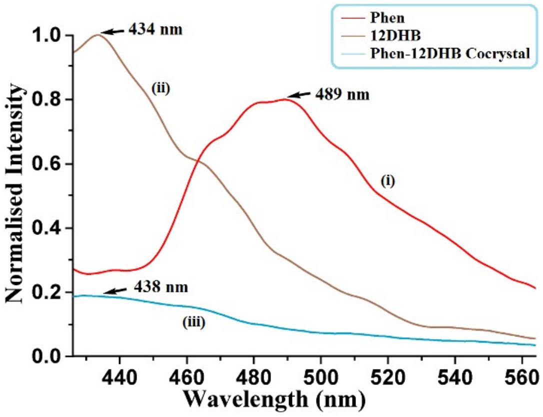

Figure 7S: Fluorescence emission of solid sample of (a) (i) Phen (λ_{ex}=355 nm, λ_{em}= 489 nm), (ii) 12DHB (λ_{ex}=365 nm, λ_{em}= 434 nm), (iii) 2(Phen).12DHB (λ_{ex}=365 nm, λ_{em}= 438 nm).

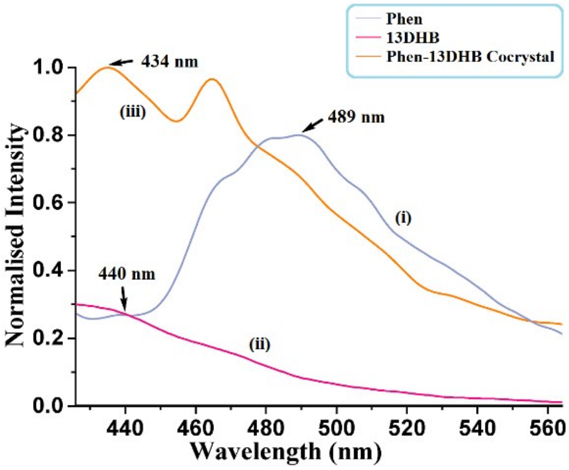

Figure 8S: Fluorescence emission of solid sample of (i) Phen (λ_{ex}=355 nm, λ_{em}= 489 nm), (ii) 13DHB (λ_{ex}=365 nm, λ_{em}= 440 nm), (iii) 2(Phen).13DHB (λ_{ex}=365 nm, λ_{em}= 434 nm).

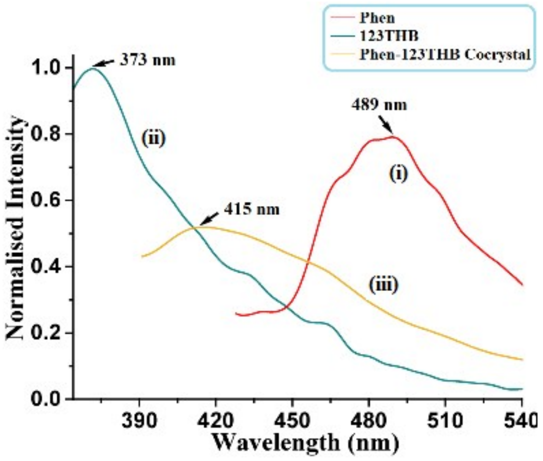

Figure 9S: Fluorescence emission of solid sample of (i) Phen (λ_{ex}=355 nm, λ_{em}= 489 nm), (ii) 123THB (λ_{ex}=355 nm, λ_{em}= 373 nm), (iii) 2.5(Phen-123).THB.2H_2O (λ_{ex}=365 nm, λ_{em}= 415 nm).

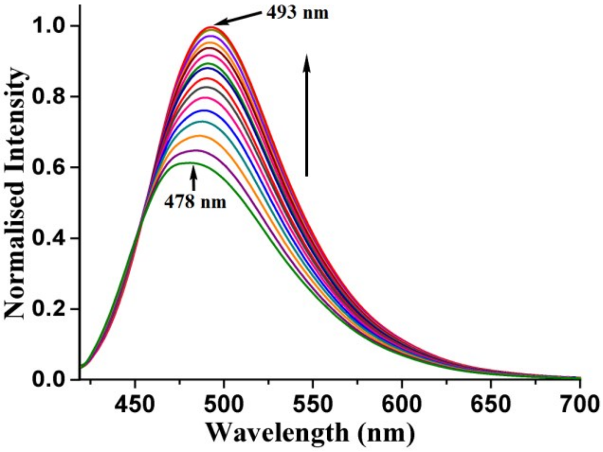

Figure 10S: Fluorescence titration (excitation at 420 nm) of Phen (10^{-3} M in acetonitrile) with 123THB (10 µL aliquot of 10^{-3} M in acetonitrile).

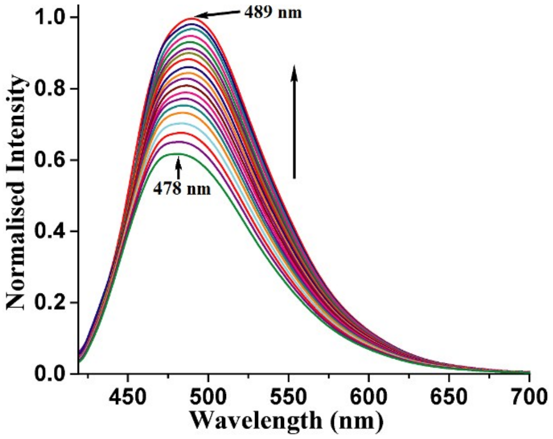

Figure 11S: Fluorescence titration (excitation at 420 nm) of phenazine (10^{-3} M in acetonitrile) with 1,3-dihydroxybenzene (10 µL aliquot of 10^{-3} M in acetonitrile).

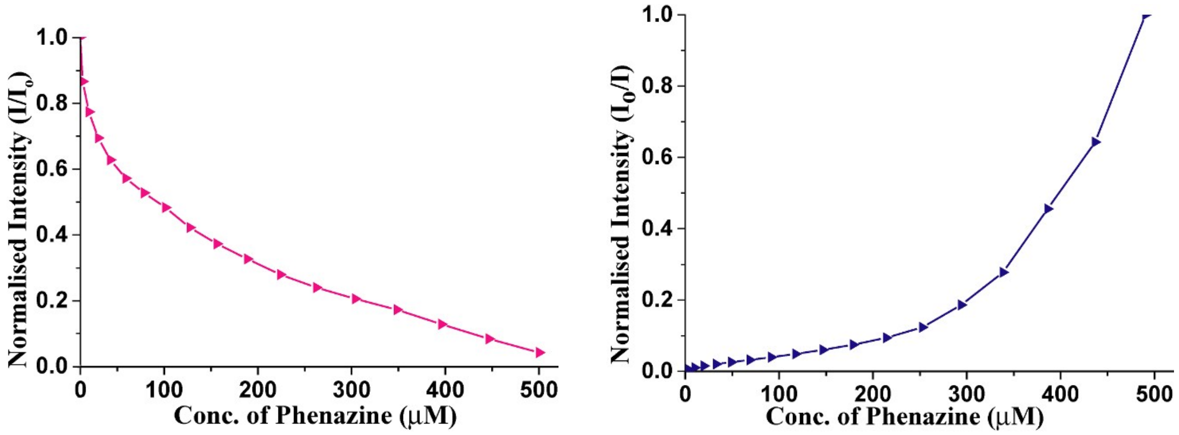

Figure 12S: (a) The changes in the fluorescence intensities at 344 nm of 2,7-dihydroxynapthalene (10^{-3} M) at different concentrations of phenazine, (b) Stern–Volmer plot from the changes in the fluorescence intensities at 344 nm of 2,7-dihydroxynaphthalene (10^{-3} M) at different concentrations of phenazine.

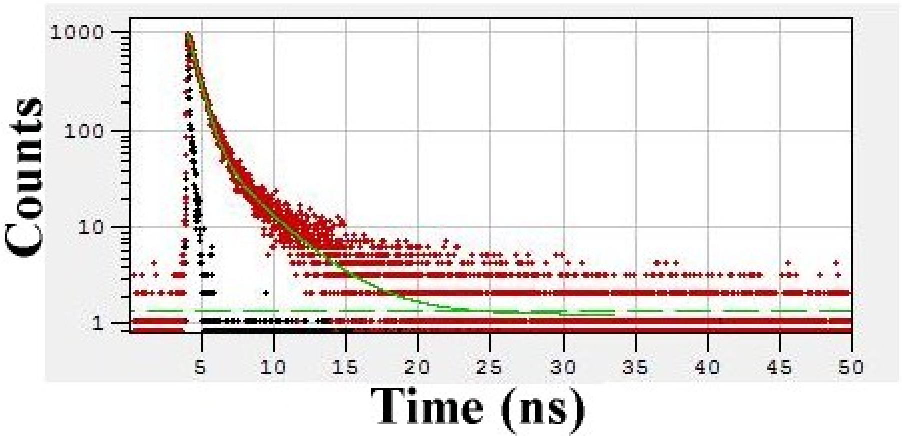

Goodness of fit χ^2 : 1.007

	B_i	ΔB_i	f_i (%)	Δf_i (%)	τ_i (ns)	$\Delta \tau_i$ (ns)
1	0.1299	0.0057	80.149	5.190	0.662	0.014
2	0.0069	0.0003	19.851	0.786	3.066	0.007

Figure 13S: Time resolved fluorescence emission of solid sample of 2(Phen).12DHB (λ_{ex}= 375 nm, λ_{em}= 438 nm).

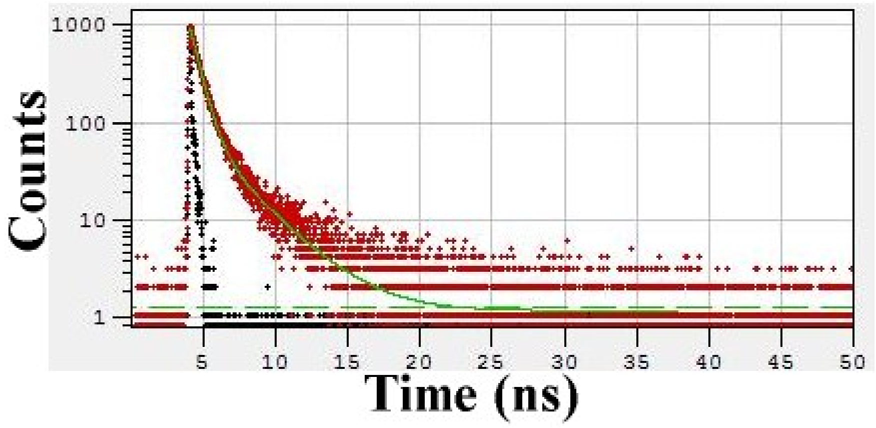

Goodness of fit χ^2: 1.009

	B_i	ΔB_i	f_i (%)	Δf_i (%)	τ_i (ns)	$\Delta \tau_i$ (ns)
1	0.1149	0.0028	78.274	3.954	0.624	0.016
2	0.0070	0.0003	21.726	0.878	2.840	0.007

Figure 14S. Time resolved fluorescence emission of solid sample of 2(Phen)13DHB (λ_{ex}= 375 nm, λ_{em}= 434 nm).

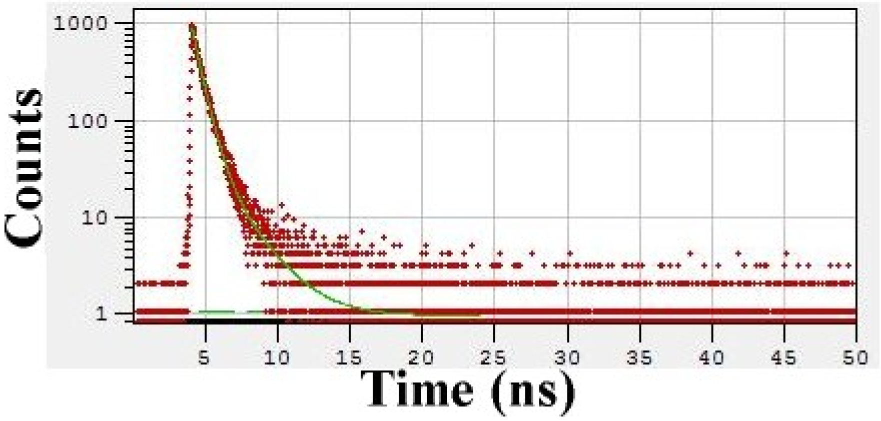

Goodness of fit χ^2 : 0.997

	B_i	ΔB_i	f_i (%)	Δf_i (%)	τ_i (ns)	$\Delta \tau_i$ (ns)
1	858.3376	4.1478	81.239	1.802	0.596	0.010
2	58.7266	1.4745	18.761	0.471	2.010 fixed	0

Figure 15S: Time resolved fluorescence emission of solid sample of 2.5(Phen).123THB $2H_2O$) (λ_{ex}= 375 nm, λ_{em}= 415 nm).

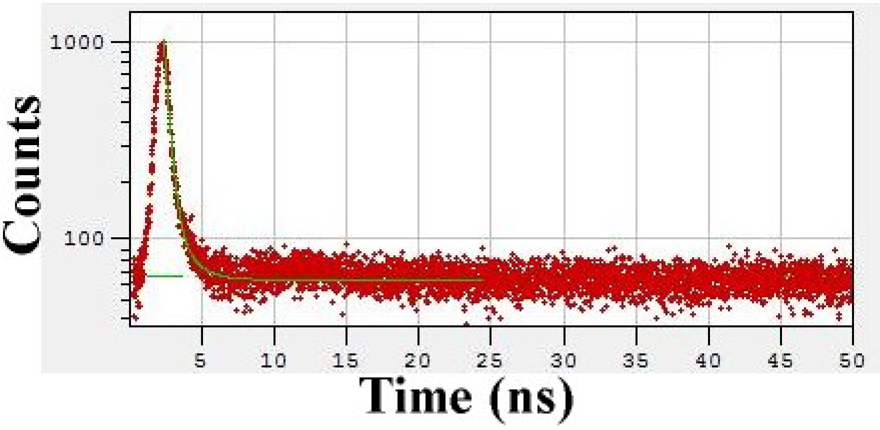

Goodness of fit χ^2 : 1.147

	B_i	ΔB_i	f_i (%)	Δf_i (%)	τ_i (ns)	$\Delta \tau_i$ (ns)
1	940.8895	19.5905	75.195	19.600	0.351	0.084
2	90.8892	21.4533	24.805	7.914	1.197	0.099

Figure 16S: Time resolved fluorescence emission of solid sample of 2.5(Phen).27DHN (λ_{ex}= 336 nm, λ_{em}= 489 nm).

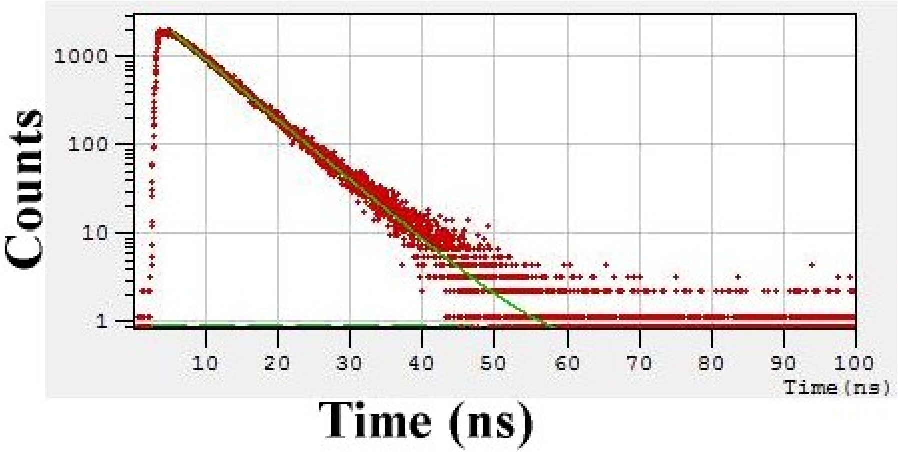

Goodness of fit χ^2: 1.010

	B_i	ΔB_i	f_i (%)	Δf_i (%)	τ_i (ns)	$\Delta \tau_i$ (ns)
1	1899.2994	3.1364	100.000	0.168	6.379	0.0002

Figure 17S: Time resolved fluorescence emission of solid sample of 27DHN (λ_{ex}= 336 nm, λ_{em}= 380 nm).

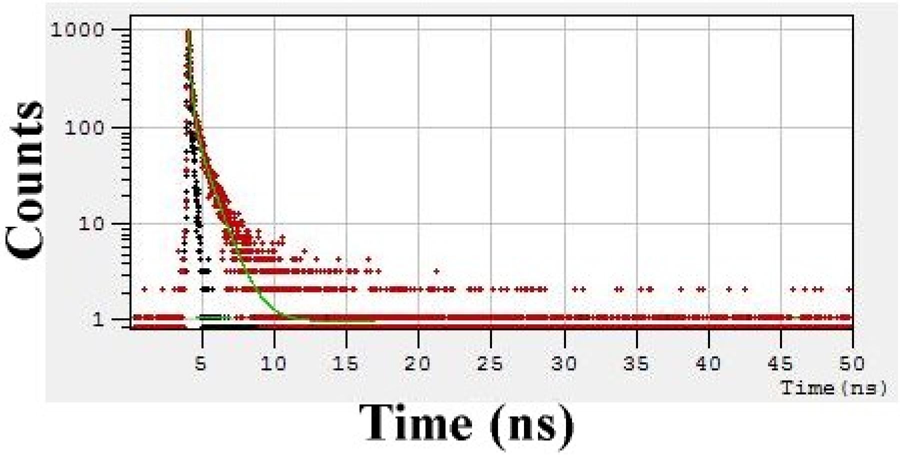

Goodness of fit χ^2: 0.999

	B_i	ΔB_i	f_i (%)	Δf_i (%)	τ_i (ns)	$\Delta \tau_i$ (ns)
1	0.2302	0.0016	64.386	0.459	0.075 fixed	0
2	0.0097	0.0002	35.614	1.112	0.988	0.013

Figure 18S: Time resolved fluorescence emission of solid sample of Phenazine (λ_{ex}= 375 nm, λ_{em}= 489 nm).

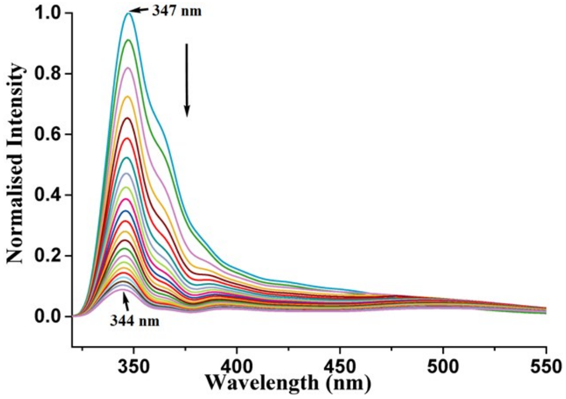

Figure 19S: Fluorescence titration (excitation at 310 nm) of 1,5-dihydroxynaphthalene (10^{-3} M in acetonitrile) by adding phenazine (10 µL aliquot of 10^{-3} M in acetonitrile).

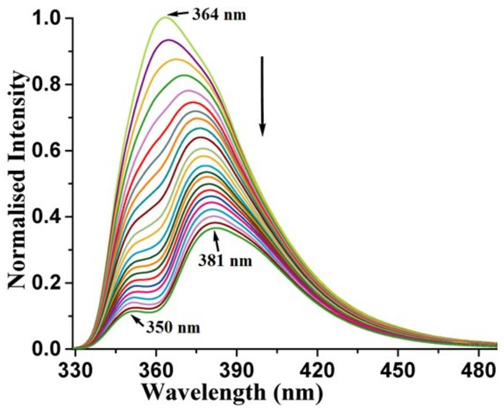

Figure 20S: Fluorescence titration (excitation at 280 nm) of 1,6-dihydroxynaphthalene (10^{-3} M in acetonitrile) by adding phenazine (10 μL aliquot of 10^{-3} M in acetonitrile).

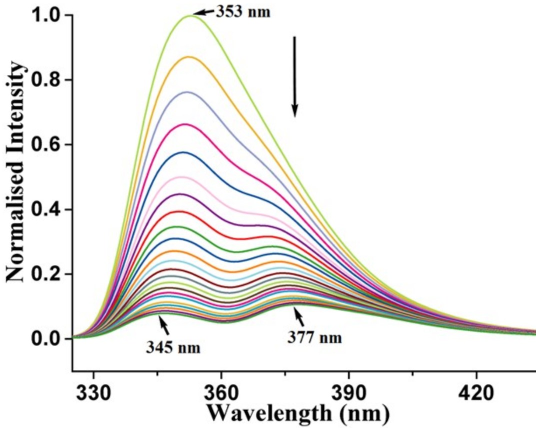

Figure 21S: Fluorescence titration (excitation at 280 nm) of 2-naphthol (10^{-3} M in acetonitrile) by adding phenazine (10 μL aliquot of 10^{-3} M in acetonitrile).

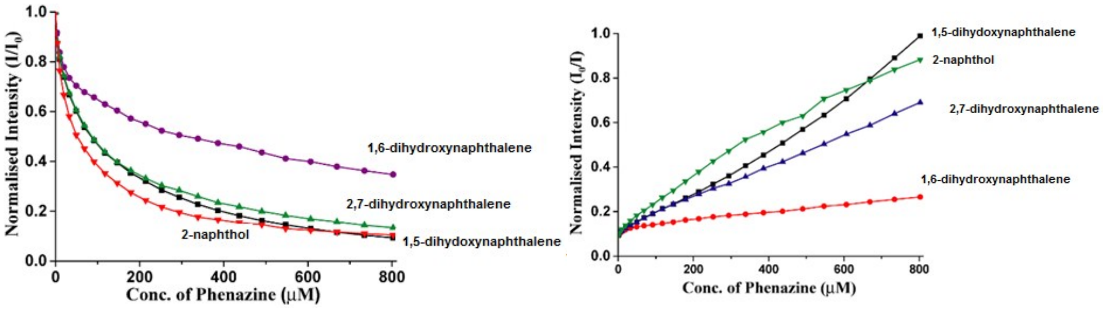

(a) (b)

Figure 22S: (a) The changes in the fluorescence intensities of different hydroxy-naphthalenes (10^{-3} M in acetonitrile) at different concentrations of phenazine and (b) Stern–Volmer plot from the changes in the fluorescence intensities of different naphthalene-ol (10^{-3} M in acetonitrile) at different concentrations of phenazine.

Table 1S: Hydrogen bond parameters of different cocrystals of phenazine

Cocrystals	D-H···A	d_{D-H} (Å)	$d_{H\cdots A}$ (Å)	$d_{D\cdots A}$ (Å)	∠D-H···A (°)
**2(Phen).12DHB**	O(1)-H(1)...N(4) [-1+x,y,z]	0.82	2.02	2.837(4)	174
	O(2)-H(2)...N(7) [-1+x,y,z]	0.82	2.04	2.843(4)	166
	O(3)-H(3)...N(1) [x, y, z]	0.82	2.09	2.902(4)	170
	O(4)-H(4)...N(6) [1-x,-y,1-z]	0.82	2.04	2.856(4)	171
	C(3)-H(3A)...N(8) [1-x,1-y,-z]	0.93	2.60	3.368(5)	140
	C(6)-H(6)...N(3) [1-x,-y,1-z]	0.93	2.54	3.316(5)	142
**2(Phen).13DHB**	O(1)-H(1)...N(4) [-1+x,y,z]	0.82	2.02	2.837(4)	174
	O(2)-H(2)...N(7) [-1+x,y,z]	0.82	2.04	2.843(4)	166
	O(3)-H(3)...N(1) [x, y, z]	0.82	2.09	2.902(4)	170
	O(4)-H(4)...N(6) [1-x,-y,1-z]	0.82	2.04	2.856(4)	171
	C(3)-H(3A)...N(8) [1-x,1-y,-z]	0.93	2.60	3.368(5)	140
	C(6)-H(6)...N(3) [1-x,-y,1-z]	0.93	2.54	3.316(5)	142
**2.5(Phen).27DHN**	O(1)-H(1)...N(3) [x,1/2-y,-1/2+z]	0.82	1.98	2.795(4)	172
	O(2)-H(2)...N(1) [1-x,-1/2+y,1/2-z]	0.82	1.96	2.766(4)	165
	C(32)-H(32)...O(2) [2-x,1/2+y,1/2-z]	0.93	2.41	3.337(5)	178
	C(33)-H(33)...N(4) [1+x,y,z]	0.93	2.58	3.491(5)	165
**2.5(Phen).123THB. 2.H_2O**	O(1)-H(1)...N(5) [-1+x,1+y,z]	0.82	2.00	2.787(3)	162
	O(2)-H(2)...O(1) [x, y, z]	0.82	2.23	2.670(3)	114
	O(2)-H(2)...O(5) [x,1+y,z]	0.82	1.94	2.675(3)	149
	O(3)-H(3)...N(2) [x, y, z]	0.82	1.96	2.757(3)	163
	O(4)-H(4A)...N(1) [x, y, z]	0.83(3)	2.06(3)	2.878(4)	170(4)
	O(4)-H(4B)...O(2) [1-x,1-y,1-z]	0.88(5)	1.81(6)	2.679(4)	168(5)
	O(5)-H(5A)...N(3) [-1+x,y,z]	0.85	2.07	2.888(5)	162
	O(5)-H(5B)...O(4) [x, y, z]	0.85	1.89	2.683(5)	155

Table 2S: Fluorescence emissions and quantum yield of phenazine and different naphthalene-ol (10^{-3}M in acetonitrile)

Naphthalene-ol	λ_{ex} (nm)	λ_{em} (nm)	Quantum Yield (Φ_F)
1,5-Dihydroxynaphthalene	310	347	0.2092
1,6-Dihydroxynaphthalene	280	364	0.3472
2,7-Dihydroxynaphthalene	280	344	0.3752
β-Naphthol	280	353	0.3805
Phenazine	420	478	0.1050
